# An adaptive microbiome α-diversity-based association analysis method

**DOI:** 10.1038/s41598-018-36355-7

**Published:** 2018-12-21

**Authors:** Hyunwook Koh

**Affiliations:** Department of Biostatistics, Johns Hopkins Bloomberg School of Public Health, Baltimore, Maryland 21205 United States

## Abstract

To relate microbial diversity with various host traits of interest (e.g., phenotypes, clinical interventions, environmental factors) is a critical step for generic assessments about the disparity in human microbiota among different populations. The performance of the current item-by-item α-diversity-based association tests is sensitive to the choice of α-diversity metric and unpredictable due to the unknown nature of the true association. The approach of cherry-picking a test for the smallest p-value or the largest effect size among multiple item-by-item analyses is not even statistically valid due to the inherent multiplicity issue. Investigators have recently introduced microbial community-level association tests while blustering statistical power increase of their proposed methods. However, they are purely a test for significance which does not provide any estimation facilities on the effect direction and size of a microbial community; hence, they are not in practical use. Here, I introduce a novel microbial diversity association test, namely, adaptive microbiome α-diversity-based association analysis (aMiAD). aMiAD simultaneously tests the significance and estimates the effect score of the microbial diversity on a host trait, while robustly maintaining high statistical power and accurate estimation with no issues in validity.

## Introduction

The human microbiome studies have been accelerated by the recent advances in high-throughput sequencing technologies^1–3^ which enabled an unbiased characterization of all microbes from different organs (e.g., gut, mouth, skin, vagina, etc.) of the human body. One of the most fundamental steps in microbiome studies is to survey the disparity in microbial diversity among different populations (e.g., case vs. control, treatment vs. placebo, or smoking vs. non-smoking). For instance, reduced microbial diversity has been found to be associated with various host phenotypes, such as obesity^4^, fatty liver disease^4^, type II diabetes^5^, inflammatory bowel diseases^6^ and additional disorders^7,8^. Clinical interventions (e.g., antibiotic use) and environmental factors (e.g., diet, smoking, delivery mode) have also been found to shift up or down the microbial diversity^9,10^. For such microbial diversity association analyses, the most commonly used approach is to relate α-diversity (within-sample microbial diversity) with a host trait of interest based on traditional statistical methods (e.g., fitting a linear regression model for the association between α-diversity and a continuous trait (e.g., body mass index (BMI)) or a logistic regression model for the association between α-diversity and a binary trait (e.g., disease/treatment status) with or without covariate adjustments). Such α-diversity-based association analysis offers systematic statistical inference facilities including the effect estimates of microbial diversity on a host trait (e.g., regression coefficient estimates) as well as hypothesis testing tools (e.g., p-values). As a result, we can comprehensively assess which population has higher or lower microbial diversity with the extent of the disparity as well as whether it is statistically significant or not.

However, many of the recent microbial community-level association tests continued to ignore some of the fundamental elements of statistical inference. For example, MiRKAT^11^, MiSPU^12^ and OMiAT^13^ produce only p-values without any effect estimation facilities (i.e., purely a test for significance). Although they boast about statistical power increase, it is difficult to lead to any novel clinical interventions or public health promotion programs based solely on p-values. To explain, suppose that we found a significant difference in a microbial community (e.g., bacterial kingdom) between diseased and healthy populations using MiRKAT, MiSPU or OMiAT. However, here, the only available conclusion is that the two populations are simply different in microbial community composition with no further understanding about how the difference exists. Instead, α-diversity-based association analysis provides effect estimation on the disparity in direction and size of the microbial diversity among different populations (e.g., the diseased population is considerably lower in microbial diversity) which are essential to better understand microbial communities (e.g., lower microbial diversity may indicate higher morbidity) and make plans (e.g., plans to recover microbial diversity to normality). In ecology, α-diversity has also been widely used as a guideline for community ecologists and conservation biologists to make plans to preserve natural ecosystems or restore perturbed communities^14–16^.

Notably, a variety of α-diversity metrics can be considered in the analysis. Different α-diversity metrics reflect different views on the true diversity and they perform differently. For example, Richness (also known as Observed), Shannon^17^ and Simpson^18^ indices are non-phylogenetic metrics (i.e., based solely on abundance information) which weight relatively rare, mid-abundant and abundant species, respectively. Accordingly, they are suitable when associated species are rare, mid-abundant and abundant species, respectively. In contrast, phylogenetic diversity (PD)^19^, phylogenetic entropy (PE)^20^ and phylogenetic quadratic entropy (PQE)^21,22^ are phylogenetic metrics (i.e., based on both abundance and phylogenetic information) which weight relatively rare, mid-abundant and abundant species, respectively. The phylogenetic metrics are suitable when associated species have disparity in both abundance and phylogeny, where PD, PE and PQE are suitable when associated species are rare, mid-abundant and abundant species, respectively. In reality, associated species can be rare or abundant, or they can have disparity in phylogeny rather than abundance or vice versa. However, it is highly difficult to predict which situation among such various possible association patterns is the one for our study and to choose a single optimal α-diversity metric to use. This is because of the unknown nature of the true association. The approach of cherry-picking a test which has the smallest p-value or the largest effect size after running multiple item-by-item α-diversity-based association analyses is not statistically valid (e.g., do not correctly control type I error) because the multiplicity (i.e., multiple testing) issue is not properly accounted for^23^. Therefore, a valid statistical method which robustly suits various unknown association patterns is needed.

In this paper, I introduce a novel adaptive microbial diversity association test, namely, adaptive microbiome α-diversity-based association analysis (aMiAD), which robustly maintains high statistical power and accurate microbial diversity effect score estimation throughout various association patterns while satisfying the requisite validity issue. aMiAD employs the minimum p-value from multiple candidate item-by-item α-diversity-based association analyses as its test statistic and estimate its own p-value and microbial diversity effect score based on a residual-based permutation method. The use of minimum p-value statistic is to adaptively approach the highest power and the most accurate microbial diversity effect score estimation among multiple candidate analyses, while the residual-based permutation method based on the minimum p-value statistic is to robustly satisfy the validity issue (e.g., correctly controlling type I error) with no distributional assumption to be satisfied. Three non-phylogenetic metrics, Richness, Shannon, Simpson indices and three phylogenetic metrics, PD, PE and PQE are selected as the candidate α-diversity metrics for aMiAD because of their distinguished features which properly modulate abundance and phylogenetic information.

The rest of the paper is organized as follows. The methodological details for aMiAD can be found in the following Methods section. Then, extensive simulations and real data applications are addressed in the Results section. I finally discuss possible extensions for the use of aMiAD in the Discussion section.

## Methods

I first organize related notations and models. Then, I address details on the six candidate α-diversity metrics, Richness, Shannon^17^, Simpson^18^, PD^19^, PE^20^ and PQE^21,22^. Finally, I delineate the test statistic and microbial diversity effect score of aMiAD and the residual permutation-based computational algorithm. While the application of aMiAD can be much broader (e.g., extendable to generalized linear models), I describe aMiAD to relate microbial diversity with a continuous (e.g., BMI) or a binary (e.g., disease/treatment status) trait.

Here, I notify that the α-diversity referred in this paper considers different types of operational taxonomic units (OTUs) in the bacterial kingdom per biological sample (e.g., human, mouse), indicating within-sample diversity of OTUs in the bacterial kingdom. However, in practice, any subunits (e.g., species or other lower-level microbial taxa) in a different microbial assemblage (e.g., kingdom of archaea, fungi, protists or viruses, phylum of firmicutes or bacteroidetes) can be considered.

### Models and notations

Suppose that there are n samples, p OTUs in a microbial community (e.g., bacterial kingdom) and q covariates (e.g., age, gender). Let Y_i_ denote a continuous (e.g., BMI) or a binary (e.g., disease/treatment status) trait, Z_ij_ denote OTUs, and X_ik_ denote covariates for i = 1, …, n, j = 1, …, p and k = 1, …, q. To relate OTUs in a community with a host trait while adjusting for covariate effects, I consider a multiple linear regression model equation (1) for a continuous trait and a multiple logistic regression model equation (2) for a binary trait.

1 $${{\rm{Y}}}_{{\rm{i}}}={\beta }_{0}+\sum _{{\rm{k}}=1}^{{\rm{q}}}\,{{\rm{X}}}_{{\rm{i}}{\rm{k}}}{\alpha }_{{\rm{k}}}+{\rm{h}}({{\rm{Z}}}_{{\rm{i}}})+{\epsilon }_{{\rm{i}}},$$

2 $${\rm{l}}{\rm{o}}{\rm{g}}{\rm{i}}{\rm{t}}\,{\rm{P}}({{\rm{Y}}}_{{\rm{i}}}=1)={\beta }_{0}+\sum _{{\rm{k}}=1}^{{\rm{q}}}\,{{\rm{X}}}_{{\rm{i}}{\rm{k}}}{\alpha }_{{\rm{k}}}+{\rm{h}}({{\rm{Z}}}_{{\rm{i}}}),$$

where β_0_ is a regression coefficient for the intercept, α_k_’s are regression coefficients for the effect of q covariates (e.g., age, gender), h (Z_i_) is a function which characterizes the relationship between OTUs and a host trait, and ∈_i_ is an error term which is independently and identically distributed with a mean zero and a variance of σ^2^. Here, we are particularly interested in testing the null hypothesis, H_0_: h (Z_i_) = 0; that is, no association between OTUs and a host trait.

Notably, we can flexibly specify h (Z_i_) to reflect different patterns of the relationship. For example, the linear relationship between OTUs and a host trait can be surveyed by setting h (Z_i_) = $\sum _{{\rm{j}}=1}^{{\rm{p}}}\,{{\rm{\beta }}}_{{\rm{j}}}{{\rm{Z}}}_{{\rm{ij}}}$, while diverse non-linear relationships can be surveyed by the use of non-linear transformations of OTUs (e.g., polynomials or splines)^24,25^. Furthermore, any positive semi-definite kernel function can be used for h (Z_i_), where MiRKAT^11^ has especially been credited with establishing a kernel machine regression framework for distance-based community-level association analysis. Among diverse alternatives, I formulate h (Z_i_) as a function of α-diversity metric equation (3) for the ultimate goal of inferring the effect of microbial diversity on a host trait.

3 $${\rm{h}}{({{\rm{Z}}}_{{\rm{i}}})}_{({\rm{\gamma }})}={{\rm{\beta }}}_{({\rm{\gamma }})}{{\rm{D}}}_{({\rm{\gamma }}){\rm{i}}},$$

where γ is an index for a chosen α-diversity metric (e.g., Richness, Shannon, Simpson, PD, PE, PQE), β(γ) is a regression coefficient for the α-diversity metric and D_(γ) i_’s are the values of the α-diversity metric for i = 1, …, n.

### α-diversity indices

α-diversity is an intuitive and natural index which summarizes the extent of microbial diversity in a community. A variety of α-diversity metrics have been proposed, and they are classified into non-phylogenetic and phylogenetic metrics. The non-phylogenetic metrics are constructed based solely on microbial abundance information, while the phylogenetic metrics further utilize phylogenetic tree information. I here survey three non-phylogenetic metrics, Richness, Shannon^17^ and Simpson^18^ indices, and three phylogenetic metrics, PD^19^, PE^20^ and PQE^21,22^.

To begin with non-phylogenetic metrics, Richness, Shannon and Simpson indices are weighted variants based on the generalized diversity framework, known as the effective number of types (or Hill number), which quantifies how many effective types of interest exist in a community^26–28^. Here, the effective number of types (D_w_) equation (4) is defined as the inverse of the mean weighted proportional abundance^26,27^.

4 $${{\rm{D}}}_{{\rm{w}}}=\frac{1}{\sqrt[{\rm{w}}-1]{{\sum }_{{\rm{j}}=1}^{{\rm{p}}}{{\rm{r}}}_{{\rm{j}}}{{\rm{r}}}_{{\rm{j}}}^{{\rm{w}}-1}}}=({\sum }_{{\rm{j}}=1}^{{\rm{p}}}{{{\rm{r}}}_{{\rm{j}}}}^{{\rm{w}}}{)}^{1/(1-{\rm{w}})},$$

where p is the total number of OTU types present in a community, r_j_ is the relative abundance (i.e., proportion) of the j-th OTU for j = 1, …, p and w ($\in {\mathbb{R}}$) is the weight for the proportions (also known as the order of the diversity) which needs to be pre-specified.

Notably, with different pre-specifications for the order of the diversity (w) equation (4), different α-diversity metrics can be derived. In particular, when w = 0, D_0_ equals to p (i.e., the total number of OTU types present in a community) which is known as Richness (D_Richness_) equation (5).

5 $${{\rm{D}}}_{{\rm{Richness}}}={{\rm{D}}}_{0}={\rm{p}},$$

where p is the total number of OTU types present in a community. When w = 1, D_1_ cannot be defined; hence, the mathematical limit of ${{\rm{l}}{\rm{i}}{\rm{m}}}_{{\rm{w}}\to 1}{{\rm{D}}}_{{\rm{w}}}=\exp (-\sum _{{\rm{j}}=1}^{{\rm{p}}}{{\rm{r}}}_{{\rm{j}}}\,{\rm{l}}{\rm{n}}\,{{\rm{r}}}_{{\rm{j}}})$^26,27^ which is the weighted geometric mean proportional abundance is alternatively employed. Then, Shannon index (D_Shannon_) equation (6) is derived by taking the logarithm to ${{\rm{l}}{\rm{i}}{\rm{m}}}_{{\rm{w}}\to 1}{{\rm{D}}}_{{\rm{w}}}$^17^.

6 $${{\rm{D}}}_{{\rm{S}}{\rm{h}}{\rm{a}}{\rm{n}}{\rm{n}}{\rm{o}}{\rm{n}}}=\,{\rm{l}}{\rm{o}}{\rm{g}}({{\rm{l}}{\rm{i}}{\rm{m}}}_{{\rm{w}}\to 1}{{\rm{D}}}_{{\rm{w}}})=-{\sum }_{{\rm{j}}=1}^{{\rm{p}}}{{\rm{r}}}_{{\rm{j}}}\,{\rm{l}}{\rm{n}}\,{{\rm{r}}}_{{\rm{j}}},$$

where p is the total number of OTU types present in a community and r_j_ is the proportion of the j-th OTU for j = 1, …, p. When w = 2, D_2_ equals to ${(\sum _{{\rm{j}}=1}^{{\rm{p}}}{{{\rm{r}}}_{{\rm{j}}}}^{2})}^{-1}$, which is the weighted arithmetic mean proportional abundance known as Inverse Simpson index^26,27^. Then, Simpson index (D_Simpson_) equation (7) is derived by taking the minus of the inverse of D_2_, −D_2_^−1^ ^18^.

7 $${{\rm{D}}}_{{\rm{Simpson}}}=-\,{{{\rm{D}}}_{2}}^{-1}=-\,\sum _{{\rm{j}}=1}^{{\rm{p}}}{{{\rm{r}}}_{{\rm{j}}}}^{2},$$

where p is the total number of OTU types present in a community and r_j_ is the proportion of the j-th OTU for j = 1, …, p.

Importantly, by the formula equation (4), we can infer that as the value of w increases, relatively abundant OTUs are weighted, but it is vice versa as the value of w decreases^27^. Therefore, Richness, Shannon and Simpson indices weight relatively rare, mid-abundant and abundant OTUs, respectively; hence, they are also suitable when associated OTUs are rare, mid-abundant and abundant, respectively.

In contrast, the phylogenetic metric, PD^19^, utilizes phylogenetic tree information while considering only the incidence (i.e., presence/absence) information of OTUs. Specifically, PD (D_PD_) is defined as the sum of the lengths of the branches for the OTUs present in a community equation (8).

8 $${{\rm{D}}}_{{\rm{PD}}}={\sum }_{{\rm{j}}=1}^{{\rm{p}}}{{\rm{l}}}_{{\rm{j}}},$$

where p is the total number of OTU types present in a community and 1_j_ is the length of all the branches that belong to the j-th OTU for j = 1, …, p. Therefore, PD is suitable when associated OTUs have high disparity in phylogeny rather than in abundance. Given that prevalent OTUs are likely to be present in all samples, PD is also suitable especially for rare OTUs which have high disparity in the classification of presence/absence.

PE^20^ equation (9) and PQE^21,22^ equation (10) are phylogenetic generalizations of the Shannon and Simpson indices, which incorporate all differing microbial abundance information (i.e., beyond the incidence (presence/absence) information for PD) while weighting relatively mid-abundant and abundant OTUs.

9 $${{\rm{D}}}_{{\rm{PE}}}=-\,\sum _{{\rm{j}}=1}^{{\rm{p}}}\,{{\rm{l}}}_{{\rm{j}}}{{\rm{r}}}_{{\rm{j}}}\,\mathrm{ln}\,{{\rm{r}}}_{{\rm{j}}},$$

10 $${{\rm{D}}}_{{\rm{PQE}}}=-\,{\sum }_{{\rm{j}}=1}^{{\rm{p}}}{{\rm{l}}}_{{\rm{j}}}{{{\rm{r}}}_{{\rm{j}}}}^{2},$$

where p is the total number of OTU types present in a community, 1_j_ is the length of all the branches that belong to the j-th OTU and r_j_ is the proportion of the j-th OTU for j = 1, …, p. Therefore, PE and PQE are suitable when associated OTUs have high disparity in phylogeny, where they are relatively mid-abundant and abundant, respectively.

The above α-diversity metrics are the most fundamental and widely used, and they were sufficient in my simulations and real data analyses. Yet, the potential extension to other α-diversity metrics is addressed later in Discussion.

### aMiAD

aMiAD is constructed based on the score test^29^ of the linear equation (1) or logistic equation (2) regression model, which surveys the association between each of the α-diversity metrics and a host trait while adjusting for covariates. Here, the unstandardized score statistic (U_(γ)_) is formulated with equation (11).

11 $${{\rm{U}}}_{({\rm{\gamma }})}={\sum }_{{\rm{i}}=1}^{{\rm{n}}}({{\rm{Y}}}_{{\rm{i}}}-{\hat{{\rm{\mu }}}}_{{\rm{i}},0}){{\rm{D}}}_{({\rm{\gamma }}){\rm{i}}}$$

where γ is an index for a chosen α-diversity metric (e.g., Richness, Shannon, Simpson, PD, PE, PQE) and ${\hat{{\rm{\mu }}}}_{{\rm{i}},0}$ is the fitted value under the null hypothesis, which is estimated as ${\widehat{{\rm{\beta }}^{\prime} }}_{0}+{\sum }_{{\rm{k}}=1}^{{\rm{q}}}{{\rm{X}}}_{{\rm{i}}{\rm{k}}}{\widehat{{\rm{\alpha }}^{\prime} }}_{{\rm{k}}}$ for the linear regression model equation (1) or ${{\rm{logit}}}^{-1}({\widehat{{\rm{\beta }}^{\prime} }}_{0}+{\sum }_{{\rm{k}}=1}^{{\rm{q}}}{{\rm{X}}}_{{\rm{ik}}}{\widehat{{\rm{\alpha }}^{\prime} }}_{{\rm{k}}})$ for the logistic regression model equation (2), where ${\widehat{{\rm{\beta }}^{\prime} }}_{0}$ and ${\widehat{{\rm{\alpha }}^{\prime} }}_{{\rm{k}}}$ are maximum likelihood estimates (MLEs) under the null hypothesis. This unstandardized score statistic (U_(γ)_) is sufficient to estimate the p-value (P_(γ)_) based on my residual permutation-based method (see Computational algorithm) because its mean and standard error are evaluated under the null hypothesis equivalently for both the observed and null (i.e., permuted) statistic values resulting in no change in their relative comparison^25^. Yet, the mean and standard error under the null hypothesis are also estimated to derive the standardized score statistic (${{\rm{U}}}_{({\rm{\gamma }})}^{\ast }$). The standardized score statistic (${{\rm{U}}}_{({\rm{\gamma }})}^{\ast }$) is asymptotically related to the regression coefficient (β_(γ)_) equation (3) and tells effect direction and size of a chosen α-diversity metric^29,30^. I denote ${{\rm{U}}}_{({\rm{\gamma }})}^{\ast }$ as MiDivES_(γ)_ and use it as the effect score of a chosen α-diversity metric.

Here, the score test equation (11) with its resulting p-value (P_(γ)_) and effect score (MiDivES_(γ)_) handles α-diversity metrics one-by-one. Yet, as described above, the performance differs according to the choice of α-diversity metric and the true underlying association pattern. Because of the unknown nature of the true association pattern, we cannot predict which α-diversity index is the optimal choice to our study in advance. Therefore, in order to robustly suit various association patterns, I propose a data-driven adaptive test, aMiAD. The test statistic of aMiAD (T_aMiAD_) is the minimum p-value from multiple item-by-item α-diversity-based association analyses equation (12).

12 $${{\rm{T}}}_{{\rm{a}}{\rm{M}}{\rm{i}}{\rm{A}}{\rm{D}}}={min}_{\gamma \epsilon {\rm{\Gamma }}}{{\rm{P}}}_{(\gamma )},$$

where γ is an index for a metric in a set of multiple candidate α-diversity metrics (Γ), where Γ = {Richness, Shannon, Simpson, PD, PE, PQE}, and P_(γ)_ is the estimated p-value for the use of each α-diversity metric (γ ∈ Γ). Here again, T_aMiAD_ equation (12) is the test statistic of aMiAD, and this minimum p-value (i.e., ${{\rm{T}}}_{{\rm{a}}{\rm{M}}{\rm{i}}{\rm{A}}{\rm{D}}}={min}_{\gamma \epsilon {\rm{\Gamma }}}{{\rm{P}}}_{(\gamma )}$ equation (12)) itself is not the p-value I report for aMiAD. The approach of cherry-picking the minimum p-value among multiple candidate analyses (i.e., ${{\rm{T}}}_{{\rm{a}}{\rm{M}}{\rm{i}}{\rm{A}}{\rm{D}}}={min}_{\gamma \epsilon {\rm{\Gamma }}}{{\rm{P}}}_{(\gamma )}$ equation (12)) and reporting it (i.e., ${{\rm{T}}}_{{\rm{a}}{\rm{M}}{\rm{i}}{\rm{A}}{\rm{D}}}={min}_{\gamma \epsilon {\rm{\Gamma }}}{{\rm{P}}}_{(\gamma )}$ equation (12)) as it is cannot correctly control type I error rates because of the inherent multiplicity (i.e., multiple testing) issue^23^. I use a residual permutation-based method (see Computational algorithm) based on the minimum p-value statistic equation (12) to estimate the p-value for aMiAD (denoted as P_aMiAD_).

The estimated microbial diversity effect score of aMiAD, namely, adaptive microbial diversity effect score (aMiDivES) equation (13), is the standardized score statistic value based on the α-diversity metric which results in the minimum p-value among multiple candidate analyses, which is then further standardized by its mean and standard error under the null hypothesis.

13 $${\rm{aMiDivES}}=\frac{{{\rm{MiDivES}}}_{({{\rm{\gamma }}}_{{\rm{m}}})}-{\rm{E}}({{\rm{MiDivES}}}_{({{\rm{\gamma }}}_{{\rm{m}}}),0})}{{\rm{SE}}({{\rm{MiDivES}}}_{({{\rm{\gamma }}}_{{\rm{m}}}),0})},$$

where γ_m_ is an index of the metric which results in the minimum p-value in a set of multiple candidate α-diversity metrics (Γ), where Γ = {Richness, Shannon, Simpson, PD, PE, PQE}, MiDivES_(γm)_ is an estimated microbial diversity effect score for the α-diversity metric which results in the minimum p-value, E(MiDivES_(γm), 0)_ and SE(MiDivES_(γm), 0)_) are the mean and standard error of MiDivES_(γm)_ under the null hypothesis. Here again, aMiDivES is the E(MiDivES_(γm)_ which is further standardized by its mean (E(MiDivES_(γm), 0_)) and standard error (SE(MiDivES_(γm), 0_)) under the null hypothesis equation (13), and the genuine microbial diversity effect score of the test reaching the minimum p-value (i.e., MiDivES_(γm)_) is not the microbial diversity effect score I report for aMiAD. I use a residual permutation-based method (see Computational algorithm) to estimate the mean (E(MiDivES_(γm), 0_)) and standard error (SE(MiDivES_(γm), 0_)).

### Computational algorithm

The computational algorithm to estimate the p-value (P_aMiAD_) and the effect score (aMiDivES) of aMiAD is based on a residual-based permutation method which randomly shuffles the residuals estimated from the null model, which reflects the null situation of no association. It is constructed based on the score statistic equation (11) and its derivatives equations (12) and (13) which do not require MLE; hence, we can avoid heavy computation and no convergence error in the iterative algorithm for MLE. It is non-parametric; hence, the outcomes are robustly valid with no underlying distributional assumption to be satisfied. The approach based on the minimum p-value statistic and a residual-based permutation method has also been widely used in prior studies^11–13,25,31^, where the validity issue was robustly satisfied. Detailed procedures can be found in (Supplementary S1 Text).

### Ethics approval and consent to participate

Not applicable. This study involves only secondary analyses. All utilized microbiome datasets are publicly and freely available which do not require any ethics approval and consent to participate.

## Results

### Simulations

I conducted simulation experiments under a wide range of scenarios in order to evaluate and compare item-by-item α-diversity-based association tests and aMiAD in terms of hypothesis testing (i.e., type I error and power) and effect score estimation (i.e., central tendency, dispersion and accuracy). I also evaluate the approach of cherry-picking a test which has the smallest p-value (denote it as Minimum P) or the largest effect size (i.e., the largest deviation from zero effect) (denote it as Largest ES) among multiple item-by-item α-diversity-based association analyses in terms of the validity issues of properly controlled type I error and the central tendency and dispersion of microbial diversity effect scores under the null hypothesis. I also evaluate other existing adaptive community-level association tests (i.e., Optimal MiRKAT (OMiRKAT)^11^, adaptive MiSPU (aMiSPU)^12^ and OMiAT^13^) in terms of hypothesis testing only (i.e., type I error and power) as they do not provide any effect estimation facilities. I applied default settings for the implementation of their software package (aMiAD ver. 1.0, MiRKAT ver. 1.0.1, MiSPU ver. 1.0, and OMiAT ver. 5.3), as suggested.

### Simulation design

I simulated microbiome data according to prior studies^11,13,25^ which reflect real OTUs’ proportions and dispersion on the basis of the Dirichlet-multinomial distribution^32^. In particular, I used real gut microbiome data^33^ from 35 fecal samples (collected from non-obese diabetic (NOD) mice at 6 weeks of age in the control group with no antibiotic treatment) for 353 OTUs (after removing OTUs with proportional mean abundance ≤10^−4^) to estimate the proportions and dispersion parameter. Then, simulation data were iteratively generated from the Dirichlet-multinomial distribution with the pre-specified values of the estimated proportions and dispersion parameter and the total reads per sample of 1,000 for small (n = 50) and large (n = 100) sample sizes, respectively^11,13,25^. Then, binary outcomes were generated based on the logistic regression model equation (14)^11,13^.

14 $${\rm{l}}{\rm{o}}{\rm{g}}{\rm{i}}{\rm{t}}\,{\rm{P}}({{\rm{y}}}_{{\rm{i}}}=1)={0.5}^{\ast }{\rm{s}}{\rm{c}}{\rm{a}}{\rm{l}}{\rm{e}}({{\rm{X}}}_{1{\rm{i}}}+{{\rm{X}}}_{2{\rm{i}}})+{\beta }^{\ast }{\sum }_{{\rm{j}}\in {\rm{\Lambda }}}{{\rm{w}}}_{{\rm{i}}}\ast {\rm{s}}{\rm{c}}{\rm{a}}{\rm{l}}{\rm{e}}({{\rm{Z}}}_{{\rm{i}}{\rm{j}}}),$$

where X_1i_ and X_2i_ are two covariates (e.g., age and gender) simulated from the normal distribution with mean 50 and standard deviation (SD) 5 and the Bernoulli distribution with success probability 0.5, respectively, β is a scalar value ($\in {\mathbb{R}}$) which determines the effect direction and size of the associated OTUs in a set Λ, where Z_ij_ is an OTU count and w_i_ is a weight for the phylogenetic disparity defined as the sum of the branch lengths for present OTUs divided by the sum of the branch lengths for absent OTUs, and ‘scale’ is the standardization function to have mean 0 and SD 1^11,13,25^. To estimate empirical type I error rate and the mean (as a measure of central tendency) and variance (as a measure of dispersion) of microbial diversity effect scores under the null hypothesis, I set β = 0. To estimate statistical power and the accuracy of effect scores, I set β from the uniform distribution between −3 and 3 (i.e., Unif(−3, 3)). Here, the R^2^ value between β values randomly generated from Unif(−3, 3) and microbial diversity effect scores estimated from each method was used as a measure of estimation accuracy. The set of associated OTUs in the community (Λ) was selected with four different scenarios: (1) Λ = {OTUs in bottom 20% in abundance}, (2) Λ = {A random 20% of OTUs}, (3) Λ = {OTUs in top 20% in abundance}, (4) Λ = {OTUs in a cluster among 7 clusters partitioned by partitioning-around-medoids (PAM) algorithm}, respectively. The first three scenarios mimic the situations when rare, mid-abundant and abundant OTUs, respectively, are associated. For the fourth scenario, I used PAM algorithm^34^ to partition all OTUs in the community into 7 clusters based on their cophenetic distances. Here, the number of clusters, 7, was selected by maximizing the average silhouette width from 5 to 10 candidate numbers of clusters^35,36^. I randomized the choice of an associated cluster among the 7 clusters to avoid arbitrary choice^13,25^, whereas the outcomes for each of the 7 clusters can be found in Supporting Information (Fig. S1). The fourth scenario mimics the situation when phylogenetically close OTUs are associated.

### Simulation results

#### Type I error

I estimate that the empirical type I error rates are well-controlled at the significance level of 0.05 for aMiAD, as well as all item-by-item α-diversity-based association tests and adaptive community-level association tests (OMiRKAT, aMiSPU and OMiAT), for both small (n = 50) and large (n = 100) sample sizes (Table 1). However, the cherry-picking approaches (i.e., Minimum P and Largest ES) show overly inflated empirical type I error rates for both small (n = 50) and large (n = 100) sample sizes (Table 1), indicating the violation of the requisite validity issue in hypothesis testing.

**Table 1:** Estimated empirical type I error rates (Unit: %).

Category	Method	n = 50	n = 100
Item-by-item α-diversity-based association tests	Richness	4.920	5.001
	Shannon	4.977	4.938
	Simpson	4.991	4.913
	PD	4.969	4.967
	PE	4.994	4.949
	PQE	4.995	4.935
Adaptive α-diversity-based association test	aMiAD	4.983	4.913
Community-level association tests	OMiRKAT	5.012	5.024
	aMiSPU	5.021	5.016
	OMiAT	5.001	5.024
Cherry-picking approaches	Minimum P	16.880	16.838
	Largest ES	16.874	16.824

Minimum P and Largest ES represent the cherry-picking approaches for the smallest p-value and the largest effect size, respectively, among multiple item-by-item α-diversity-based association analyses.

#### Central tendency and dispersion of effect scores under the null hypothesis

I estimate that the means of microbial diversity effect scores under the null hypothesis are around zero, indicating no bias in the estimation, for all surveyed tests and for both small (n = 50) and large (n = 100) sample sizes (Table 2). I also estimate that the variances of microbial diversity effect scores under the null hypothesis are around one for aMiAD, as well as all the item-by-item α-diversity-based association tests, for both small (n = 50) and large (n = 100) sample sizes (Table 2). However, the cherry-picking approaches (i.e., Minimum P and Largest ES) show overly inflated variance estimates for both small (n = 50) and large (n = 100) sample sizes (Table 2), indicating over-estimation of effect size.

**Table 2:** Estimated means and variances of microbial diversity effect scores under the null hypothesis (Unit: %).

Category	Method	n = 50		n = 100	
		Mean	Variance	Mean	Variance
Item-by-item α-diversity-based association tests	Richness	−0.004	1.008	0.000	1.004
	Shannon	−0.001	1.007	−0.003	1.004
	Simpson	−0.001	1.007	−0.002	1.003
	PD	−0.002	1.004	0.000	0.997
	PE	−0.002	1.005	−0.003	0.996
	PQE	−0.001	1.010	−0.003	0.998
Adaptive α-diversity-based association test	aMiAD	−0.002	1.004	−0.001	1.001
Cherry-picking approaches	Minimum P	−0.006	2.285	−0.001	2.285
	Largest ES	−0.002	2.287	0.000	2.287

Minimum P and Largest ES represent the cherry-picking approaches for the smallest p-value and the largest effect size, respectively, among multiple item-by-item α-diversity-based association analyses.

#### Power and estimation accuracy

**Figure 1:**
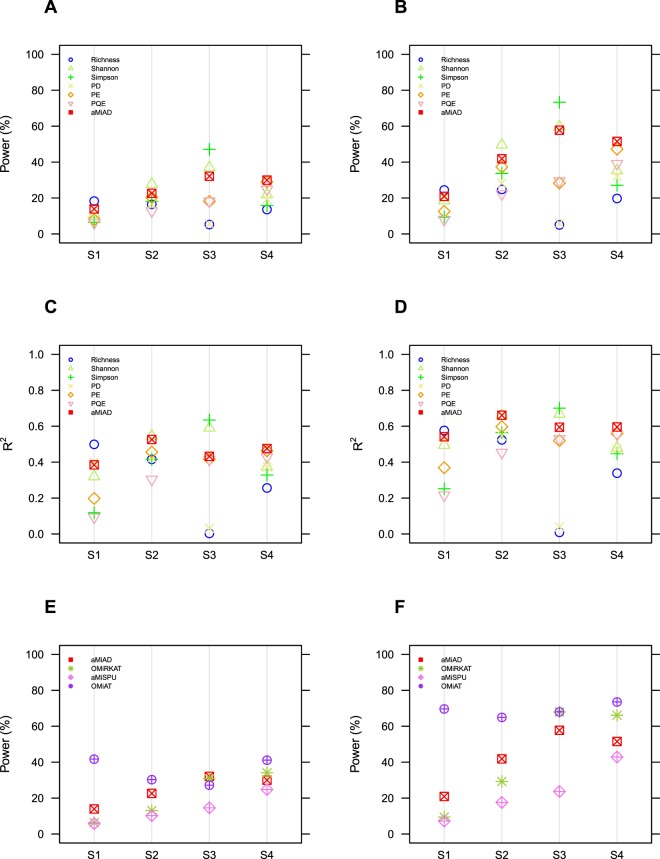
Estimated powers and R^2^ values. (**A**) Estimated powers for the α-diversity-based association tests for the scenarios, where rare (S1), mid-abundant (S2), abundant (S3) and phylogenetically close (S4) OTUs are associated (n = 50). (**B**) Estimated powers for the α-diversity-based association tests for the scenarios, where rare (S1), mid-abundant (S2), abundant (S3) and phylogenetically close (S4) OTUs are associated (n = 100). (**C**) Estimated powers for the adaptive association tests for the scenarios, where rare (S1), mid-abundant (S2), abundant (S3) and phylogenetically close (S4) OTUs are associated (n = 50). (**D**) Estimated powers for the adaptive association tests for the scenarios, where rare (S1), mid-abundant (S2), abundant (S3) and phylogenetically close (S4) OTUs are associated (n = 100). (**E**) Estimated R^2^ values for the α-diversity-based association tests for the scenarios, where rare (S1), mid-abundant (S2), abundant (S3) and phylogenetically close (S4) OTUs are associated (n = 50). (**F**) Estimated R^2^ values for the α-diversity-based association tests for the scenarios, where rare (S1), mid-abundant (S2), abundant (S3) and phylogenetically close (S4) OTUs are associated (n = 100). **S1. Λ = {OTUs in bottom 20% in abundance}; S2. Λ = {A random 20% of OTUs}; S3. Λ = {OTUs in top 20% in abundance}; S4. Λ = {OTUs in a random cluster (average) among the 7 clusters partitioned by PAM}.

To begin with comparing the performance of α-diversity-based association tests, Richness estimates the greatest power and R^2^ values when rare OTUs are associated for both small (n = 50) (Figs 1A,C and (S1)) and large (n = 100) (Figs 1B,D and (S1)) sample sizes, while the Shannon index estimates the greatest power and R^2^ values when mid-abundant OTUs are associated for both small (n = 50) (Figs 1A,C and (S2)) and large (n = 100) (Figs 1B,D and (S2)) and the Simpson index estimates the greatest power and R^2^ values when abundant OTUs are associated for both small (n = 50) (Figs 1A,C and (S3)) and large (n = 100) (Figs 1B,D and (S3)), which are explained by their abundance weighting schemes. When phylogenetically close OTUs are associated (i.e., OTUs in a random cluster among the 7 clusters partitioned by the PAM algorithm are associated), the phylogenetic metrics (i.e., PD, PE and PQE) estimates greater power and R^2^ values than the non-phylogenetic metrics (i.e., Richness, Shannon and Simpson) for both small (n = 50) (Figs 1A,C and (S4)) and large (n = 100) (Figs 1B,D and (S4)) sample sizes, where PE estimates the greatest power and R^2^ values. This is because the phylogenetic metrics further incorporate phylogenetic information, while the non-phylogenetic metrics are based only on abundance information. To be more detailed, the performance also varies by which cluster among the 7 clusters partitioned by PAM algorithm is selected (see Supporting Information (Fig. S1)). That is, the Shannon index estimates the greatest power and R^2^ values when OTUs in the first cluster are associated (Fig. S1A–D(C1)), PE estimates the greatest power and R^2^ values when OTUs in the second, third, fifth and sixth clusters are associated (Fig. S1A–D(C2, C3, C5, C6)), and PQE estimates the greatest power and R^2^ values when OTUs in the fourth cluster are associated (Fig. S1A–D(C4, C7)).

Although it may not be feasible to reflect all possible true association patterns in the natural world to our simulations, the most meaningful observation here is that aMiAD adaptively approaches the greatest power and R^2^ values among different item-by-item analyses throughout all surveyed scenarios (Figs 1A–D and S1A–D), while the performance for each α-diversity metric considerably fluctuates (Figs 1A–D and S1A–D). In reality, the true association scenario is mostly unknown, while a variety of scenarios are also likely to exist. Thus, aMiAD is attractive due to its high adaptivity and robustness to better cope with the unknown nature.

To compare aMiAD with the three adaptive community-level association tests (OMiRKAT, aMiSPU and OMiAT) (Figs 1E,F and S1E,F), OMiAT estimates the greatest power values for most of the scenarios except that aMiAD estimates the greatest power values for small sample size (n = 50) when abundant OTUs (Figs 1E and (S3)) and OTUs in the second cluster among the 7 clusters partitioned by the PAM algorithm are associated (Fig. S1E(C2)), aMiSPU estimates the greatest power values when OTUs in the fourth cluster are associated for both small (n = 50) (Fig. S1E(C4)) and large (n = 100) (Fig. S1F(C4)) sample sizes and OMiRKAT estimates the greatest power values when OTUs in the seventh cluster are associated for both small (n = 50) (Fig. S1E(C7)) and large (n = 100) (Fig. S1F(C7)) sample sizes. To summarize, we may conclude that OMiAT is most robustly powerful. However, once again, OMiAT, as well as OMiRKAT and aMiSPU, does not provide any effect estimation facilities; hence, its interpretability and usability are limited.

### Real data applications

#### The disparity in microbial diversity between control and antibiotic treatment groups

Cox *et al*. (2013) performed microbiota-profiling studies to survey if the gut microbiota affected during maturity by antibiotic treatment leads to continued metabolic consequences^37^. To demonstrate the use of aMiAD, I analyzed a part of the original data, which surveys the effect of antibiotic treatment with low-dose penicillin (LDP) on microbial diversity of the gut microbiota. In particular, I compared microbial diversity of the bacterial kingdom between two groups of mice, 8 control and 7 antibiotic treatment mice. To summarize the sampling and profiling procedures while details are found in the original literature^37^, the 8 control mice are 8 germ-free mice to whom cecal microbiota from mice with no treatment were transferred and the 7 antibiotic treatment mice are 7 germ-free mice to whom cecal microbiota from LDP-treated mice were transferred. Fecal samples from the 8 control and 7 antibiotic treatment mice were collected after 23 days of the transfer, and the V4 region of the bacterial 16S rRNA gene was targeted in the amplicon sequencing with barcoded fusion primers^38^. Then, the QIIME pipeline^2^ was used to quantify OTUs and construct their phylogenetic tree. The OTUs were rarefied using the software package, phyloseq^39^ due to the varying total reads per sample^40^. 59 OTUs were included in the analysis after removing OTUs which are not present in any sample after random subsampling of the rarefaction^39^. Here, only a few OTUs (i.e., 59 OTUs), which may not represent the entire ecosystem, were analyzed because of some data quality issues (e.g., small sample size, low sequencing depth and the antibiotic treatment effect which can substantially reduce microbial abundance/diversity).

**Figure 2:**
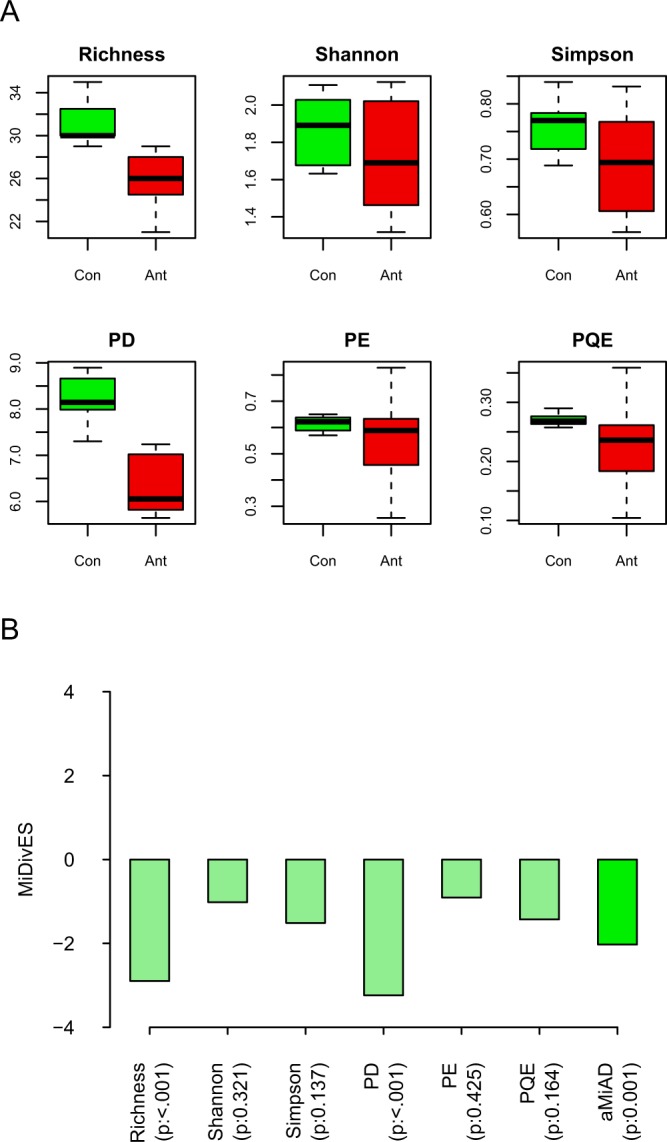
The comparison between the control (Con) and antibiotic treatment (Ant) groups. (**A**) The box-plots for each α-diversity metric (i.e., Richness, Shannon, Simpson, PD, PE, PQE). (**B**) The outcomes for the item-by-item (i.e., Richness, Shannon, Simpson, PD, PE, PQE) and adaptive (i.e., aMiAD) α-diversity-based association tests.

We can first visually observe in the box-plots (Fig. 2A) that all the α-diversity metrics are lower for the antibiotic treatment group than the control group, while PD and then Richness show the greatest disparity. Correspondingly, we can observe negative estimated effect scores for all α-diversity metrics, indicating microbial diversity is lower for the antibiotic treatment group than the control group, where the disparity is especially significant for PD (p-value: <0.001) and Richness (p-value: <0.001) indices (Fig. 2B). aMiAD estimates that microbial diversity is significantly different between the two groups (p-value: 0.001), where the microbial diversity is lower for the antibiotic treatment group than the control group (aMiDivES: −2.028 < 0) (Fig. 2B).

#### The disparity in microbial diversity between non-diseased and diseased groups

Environmental exposures (e.g., antibiotic use) during maturation have been associated with immunological and metabolic development through the mechanisms involved in the interaction between microbiota and host^41^. Type 1 diabetes (T1D) is one of the most common autoimmune diseases, which is caused by pancreatic β-cell destruction. T1D often appears in the pediatric age, and its incidence rate is globally increasing^42^. Livanos *et al*., (2016) performed microbiota-profiling studies to survey if the gut microbiota mediates the effect of antibiotic treatment on T1D onset^33^. To demonstrate the use of aMiAD, I analyzed a part of the original data, which surveys if the microbial diversity of gut microbiota altered by antibiotic treatment is differential by T1D status. To summarize the sampling and profiling procedures^33^, 19 NOD mice were exposed to the antibiotic (specifically, therapeutic-dose pulsed antibiotic) treatment, then, their fecal samples were collected after 6 weeks of the exposure. The V4 region of the bacterial 16S rRNA gene was targeted in the amplicon sequencing with barcoded fusion primers^38^ and the QIIME pipeline^2^ was used to quantify OTUs and construct their phylogenetic tree. The OTUs were rarefied using the software package, phyloseq^39^ due to the varying total reads per sample^40^. 390 OTUs were included in the analysis after removing OTUs which are not present in any sample after random subsampling of the rarefaction^39^.

**Figure 3:**
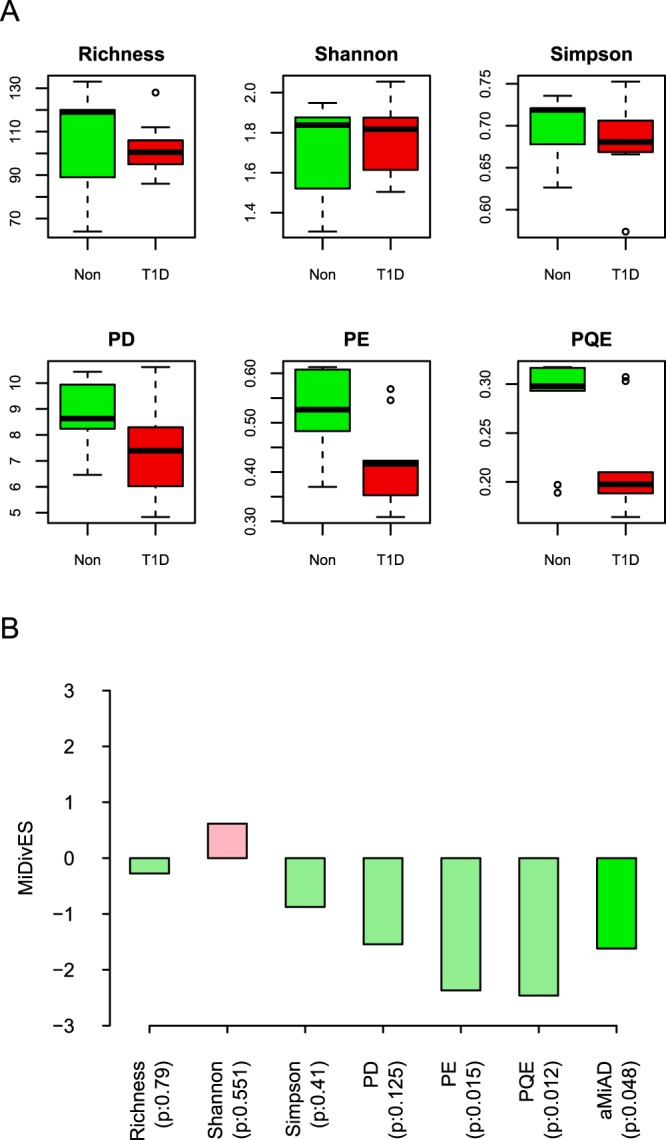
The comparison between the non-diseased (Non) and diseased (T1D) groups. (**A**) The box-plots for each α-diversity metric (i.e., Richness, Shannon, Simpson, PD, PE, PQE). (**B**) The outcomes for the item-by-item (i.e., Richness, Shannon, Simpson, PD, PE, PQE) and adaptive (i.e., aMiAD) α-diversity-based association tests.

We can first visually observe in the box-plots (Fig. 3A) that the phylogenetic metrics (PD, PE and PQE) show a greater disparity than the non-phylogenetic metrics (Richness, Shannon and Simpson), where PQE and then PE show the greatest disparity. Here, we can also observe that the microbial diversity is lower for the T1D group than the non-diseased group for all α-diversity metrics but the Shannon index (Fig. 3A). Correspondingly, PQE (p-value: 0.012) and PE (p-value: 0.015) estimate significant p-values with negative effect direction (Fig. 3B). The Shannon index is the only metric which estimates positive effect direction (Fig. 3B). This indicates that item-by-item analyses are substantially sensitive to (e.g., the decision on significance and/or effect direction can even be reversed by) the choice of α-diversity metric. aMiAD estimates that microbial diversity is significantly different between the two groups (p-value: 0.048), where the microbial diversity is lower for the T1D group than the non-diseased group (aMiDivES: −1.619 < 0) (Fig. 3B).

## Discussion

The recent microbial community-level association tests might be more powerful, where we, especially, observed in Simulations that OMiAT is most robustly powerful (Figs 1E,F and S1E,F). However, they do not provide any effect estimation facilities; hence, any further information about the disparity in microbial community composition is not accessible. Instead, aMiAD additionally estimates microbial diversity effect score, which can further enhance the interpretability. Here, I briefly discuss that other ANOVA-based methods (e.g., mvabund^43^) cannot directly adjust potential confounding effects (e.g., age, gender), while the regression-based methods (e.g., MiRKAT, MiSPU, OMiAT, aMiAD) can easily adjust them.

I chose the six α-diversity metrics, Richness, Shannon^17^, Simpson^18^, PD^19^, PE^20^ and PQE^21,22^, as the candidate α-diversity metrics for aMiAD because of their distinguished features^44^. However, we are not restricted to these metrics, and other α-diversity metrics might be considered. For example, Chao1^45^ and ACE^46^, can be used to further modulate the extent of the rarity of association OTUs. Chao1 and ACE utilize abundance information as “≥2 or <2 reads” and “≥10 or <10 reads”, respectively, while Richness utilizes it as presence (i.e., ≥1 reads) or absence (i.e., 0 read). Thus, we may expect that Chao1 might be suitable when the extent of the rarity is relatively lower than the one for Richness, but relatively higher than the one for ACE. The Inverse Simpson index can also be considered by replacing the original Simpson index. Yet, I heuristically determined to use the original Simpson index as the Inverse Simpson index did not show any better performance. Notably, novel statistical estimates for α-diversity have still been proposed while further addressing the issues of missing species, sampling noise, experimental noise and so forth^47–52^. Any α-diversity metrics can be easily employed in my software package, aMiAD, through user options.

In this paper, I introduced aMiAD which adaptively approaches to the highest power and the most accurate microbial diversity effect score estimation among multiple item-by-item α-diversity-based association analyses. aMiAD also robustly satisfies the requisite validity issues in hypothesis testing and effect score estimation. Although I proposed aMiAD to relate microbial diversity with a continuous (e.g., BMI) or binary (e.g., disease/treatment status) trait of interest, it would be extendable to different types of trait (e.g., survival, multinomial trait)^25,53–55^. Moreover, an extension to the linear mixed effect model^56^/generalized linear mixed effect model^57^ is needed for correlated (e.g., family-based or longitudinal) study designs.

## Electronic supplementary material

Supplementary Information

## Data Availability

The utilized microbiome data are publicly available at the European Bioinformatics Institute (EBI) database (https://www.ebi.ac.uk, accession code: ERP016357)^33^ and the Sequence Read Archive (SRA) repository (https://www.ncbi.nlm.nih.gov/sra, accession code: SRP042293)^37^. The software package, aMiAD, is freely available at https://github.com/hk1785/aMiAD.
