# Supplementary material for: An adaptive microbiome α-diversity-based association analysis method
Source: Sci Rep. 2018 Dec 21;8:18026. doi: 10.1038/s41598-018-36355-7 (PMC6303306; doi:10.1038/s41598-018-36355-7)
Supplement: Supplementary file 1 — Supplementary Information [file 41598_2018_36355_MOESM1_ESM.pdf]

Supplementary Information

An adaptive microbiome α-diversity-based association analysis method

Hyunwook Koh

Department of Biostatistics, Johns Hopkins Bloomberg School of Public Health,

Baltimore, Maryland 21205, United States

Email: hkoh7@jhu.edu

Text S1. Computational procedures

1. Fit the null model $\hat{\mu}_{i,0} = \widehat{\beta'}_0 + \sum_{k=1}^{q} X_{ik}\, \widehat{\alpha'}_k$ for the linear regression model or $\hat{\mu}_{i,0} = \text{logit}^{-1}(\widehat{\beta'}_0 + \sum_{k=1}^{q} X_{ik}\, \widehat{\alpha'}_k)$ for the logistic regression model and denote the residuals as $R_i = Y_i - \hat{\mu}_{i,0}$, i = 1, …, n.
2. Calculate the observed unstandardized score statistic value $U_{(\gamma)} = \sum_{i=1}^{n} R_i\, D_{(\gamma)i}$ for each metric in Γ equation (11).
3. Permute the residuals R_i, i = 1, …, n and denote the permuted residuals as R'_i, i = 1, …, n. Repeat this procedure multiple times (say, G times) and denote the permuted residuals for each iteration as $R'_{(g),i}$, i = 1, …, n, where g is an index for each iteration, g ∊ {1, ..., G}.
4. Calculate the null (permuted) unstandardized score statistic values $U_{(\gamma,g)} = \sum_{i=1}^{n} R'_{(g),i}\, D_{(\gamma)i}$ for each metric in Γ and for each iteration, g ∊ {1, …, G} equation (11).
5. Calculate the p-value $P_{(\gamma)} = \sum_{g=1}^{G}[I(|U_{(\gamma,g)}| \geq |U_{(\gamma)}|)]/G$ for each metric in Γ, where $I(\cdot)$ is an indicator function.
6. Calculate the standardized score statistic value $\text{MiDivES}_{(\gamma)} = \frac{U_{(\gamma)} - \text{Mean}(\{U_{(\gamma,1)},\ldots,U_{(\gamma,G)}\})}{\text{SD}(\{U_{(\gamma,1)},\ldots,\{U_{(\gamma,G)}\})}$ for each metric in Γ.
7. Calculate the observed unstandardized statistic value $T_{aMiAD} = \min_{\gamma \epsilon \Gamma} P_{(\gamma)}$ equation (12).
8. Calculate the null (permuted) unstandardized statistic values $T_{aMiAD(g)} = \min_{\gamma \epsilon \Gamma}\{\sum_{g' \neq g}[I(T_{aMiAD(g')} \geq T_{aMiAD(g)})]/(G\text{-}1)\}$, where g and g′ are an index for each iteration, g ∊ {1, ..., G} and g′ ∊ {1, ..., G}.
9. Calculate the p-value $P_{aMiAD} = \sum_{g=1}^{G}[I(T_{aMiAD(g)} \leq T_{aMiAD})]/G$.
10. Calculate the standardized statistic value aMiDivES = $\frac{\text{MiDivES}_{(\gamma_m)} - \text{Mean}(\{T_{aMiAD(1)},\ldots,T_{aMiAD(G)}\})}{\text{SD}(\{T_{aMiAD(1)},\ldots,T_{aMiAD(G)}\})}$ equation (13).

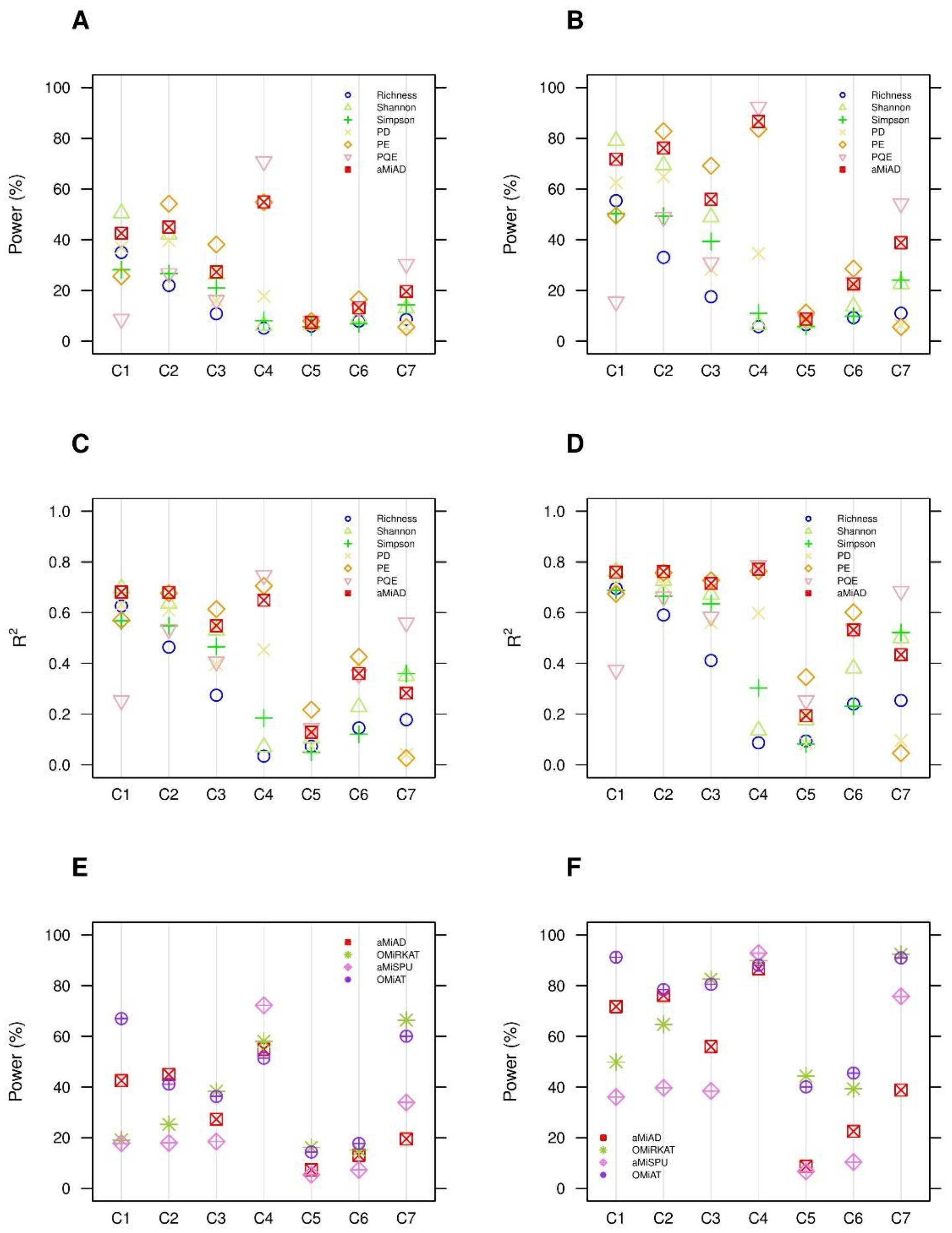

S1 Figure. Estimated powers and R^2 values **A.** Estimated powers for the α-diversity-based association tests for each selected cluster (C1-C7) among the 7 clusters partitioned by PAM (n=50). **B.** Estimated powers for the α-diversity-based association tests for each selected cluster (C1-C7) among the 7 clusters partitioned by PAM (n=100). **C.** Estimated powers for the adaptive association tests for each selected cluster (C1-C7) among the 7 clusters partitioned by PAM (n=50). **D.** Estimated powers for the adaptive association tests for each selected cluster (C1-C7) among the 7 clusters partitioned by PAM (n=100). **E.** Estimated R^2 values for the α-diversity-based association tests for each selected cluster (C1-C7) among the 7 clusters partitioned by PAM (n=50). **F.** Estimated R^2 values for the α-diversity-based association tests for each selected cluster (C1-C7) among the 7 clusters partitioned by PAM (n=100). ** **C1**. Λ = {130 OTUs in the first cluster (13.8% of total abundance)}; **C2.** Λ = {81 OTUs in the second cluster (15.5% of total abundance)}; **C3.** Λ = {52 OTUs in the third cluster (15.5% of total abundance)}; **C4.** Λ = {15 OTUs in the fourth cluster (33.3% of total abundance)}; **C5.** Λ = {14 OTUs in the fifth cluster (1.1% of total abundance)}; **C6.** Λ = {18 OTUs in the sixth cluster (1.8% of total abundance)}; **C7.** Λ = {43 OTUs in the seventh cluster (19.0% of total abundance)}.
